# High-Throughput Screening of Type III Secretion Determinants Reveals a Major Chaperone-Independent Pathway

**DOI:** 10.1128/mBio.01050-18

**Published:** 2018-06-19

**Authors:** Nadja Heinz Ernst, Analise Z. Reeves, Julia E. Ramseyer, Cammie F. Lesser

**Affiliations:** a Department of Medicine, Division of Infectious Diseases, Massachusetts General Hospital, Cambridge, Massachusetts, USA; b Department of Microbiology and Immunobiology, Harvard Medical School, Boston, Massachusetts, USA; c Broad Institute of MIT and Harvard, Cambridge, Massachusetts, USA; Harvard Medical School

**Keywords:** *Shigella*, chaperone, sorting platform, type III secretion system

## Abstract

Numerous Gram-negative bacterial pathogens utilize type III secretion systems (T3SSs) to inject tens of effector proteins directly into the cytosol of host cells. Through interactions with cognate chaperones, type III effectors are defined and recruited to the sorting platform, a cytoplasmic component of these membrane-embedded nanomachines. However, notably, a comprehensive review of the literature reveals that the secretion of most type III effectors has not yet been linked to a chaperone, raising questions regarding the existence of unknown chaperones as well as the universality of chaperones in effector secretion. Here, we describe the development of the first high-throughput type III secretion (T3S) assay, a semiautomated solid-plate-based assay, which enables the side-by-side comparison of secretion of over 20 *Shigella* effectors under a multitude of conditions. Strikingly, we found that the majority of *Shigella* effectors are secreted at equivalent levels by wild-type and variants of *Shigella* that no longer encode one or all known *Shigella* T3S effector chaperones. In addition, we found that *Shigella* effectors are efficiently secreted from a laboratory strain of Escherichia coli expressing the core *Shigella* type III secretion apparatus (T3SA) but no other *Shigella-*specific proteins. Furthermore, we observed that the sequences necessary and sufficient to define chaperone-dependent and -independent effectors are fundamentally different. Together, these findings support the existence of a major, previously unrecognized, noncanonical chaperone-independent secretion pathway that is likely common to many T3SSs.

## IMPORTANCE

Many bacterial pathogens use specialized nanomachines, including type III secretion systems, to directly inject virulence proteins (effectors) into host cells. Here, we present the first extensive analysis of chaperone dependence in the process of type III effector secretion, providing strong evidence for the existence of a previously unrecognized chaperone-independent pathway. This noncanonical pathway is likely common to many bacteria, as an extensive review of the literature reveals that the secretion of multiple type III effectors has not yet been knowingly linked to a chaperone. While additional studies will be required to discern the molecular details of this pathway, its prevalence suggests that it can likely serve as a new target for the development of antimicrobial agents.

## INTRODUCTION

Numerous Gram-negative bacteria, including *Shigella*, *Salmonella*, *Escherichia*, and *Yersinia* species, utilize type III secretion systems (T3SSs) to inject proteins directly into host cells. T3SSs are complex nanomachines composed of 20 to 25 different proteins that form a membrane-embedded needle complex (1). Upon contact with host cells, the protein complex at the tip of the needle, the translocon, forms a pore in the host cell membrane, completing the channel that serves as a conduit for the delivery (translocation) of effector proteins into the host cell cytosol. These effectors proceed to usurp host cellular processes to promote bacterial replication and spread. While each pathogen encodes its own unique set of effectors, many of the structural components of their type III secretion apparatuses (T3SAs) are conserved, suggesting a common mechanism of effector recognition.

On the basis of studies primarily conducted in the 1990s, type III secreted (T3S) effectors are currently typically described as containing a bipartite secretion signal composed of an extreme amino-terminal secretion sequence followed by a chaperone-binding domain (CBD) within their first 50 to 100 residues (2–4). The N-terminal secretion sequence is not defined but rather is characterized by its intrinsically disordered nature (5), and while essential, its role in secretion remains to be discovered. In contrast, there is evidence that structural motifs common to chaperone-effector complexes serve as the three-dimensional signals that define effectors and target their recognition by the T3SA (4, 6, 7) Furthermore, through interactions with cognate chaperones, effectors are recruited to the sorting platform, the multiprotein complex that docks on the cytoplasmic surface of the membrane-embedded T3SA (8–11).

And yet, a comprehensive review of the literature reveals that over the past 20 years chaperones have been identified for only a third (38/109) of the effectors of the well-studied *Shigella* Mxi-Spa (11/31), *Salmonella* SPI1 (6/11), *Salmonella* SPI2 (4/20), and *Yersinia* Ysc (4/6) as well as enteropathogenic Escherichia coli/enterohemorrhagic E. coli (EPEC/EHEC) Esc (13/41) T3SSs (12–19) (see Table S1 in the supplemental material). These observations question the commonly held notion that chaperones play essential roles in effector secretion and raise the possibility of the existence of unidentified chaperones and/or a chaperone-independent T3S pathway.

10.1128/mBio.01050-18.5 TABLE S1  Summary of effectors and their cognate chaperones. Download TABLE S1, DOCX file, 0.01 MB. Copyright © 2018 Ernst et al. 2018 Ernst et al. This content is distributed under the terms of the Creative Commons Attribution 4.0 International license.

Here we describe the development of a semiautomated solid-plate-based secretion assay to study the secretion of Shigella flexneri effectors. This high-throughput type III secretion assay enabled the first comprehensive investigation of the roles of known and candidate T3S chaperones in mediating effector secretion. Using this assay, in addition to confirming all previously established *Shigella* effector chaperone dependencies, we determined that the majority of *Shigella* effectors are efficiently secreted independently of all known and numerous candidate T3S chaperones. Furthermore, we found that, in contrast to chaperone-dependent effectors, the sequences that define chaperone-independent effectors are not restricted to their amino termini but rather are located throughout the effector. Together, these findings strongly suggest the existence of a major, previously unappreciated, T3S chaperone-independent type III effector secretion pathway, likely common to multiple pathogens.

## RESULTS

### A solid-plate-based assay increases throughput of detection of *Shigella* type III effector secretion.

Upon contact with host cells, the translocon complex, positioned at the tip of the T3SA, is inserted into the host membrane. This interaction triggers a conformational change, which leads to activation of the T3SA resulting in the injection of translocon components followed by effectors into host cells. *In vitro* conditions that mimic host cell contact and trigger secretion into liquid media have been established for several pathogens (20, 21). In the case of *Shigella* species, exposure of liquid exponential-phase cultures to the dye Congo red (CR) stimulates type III secretion activation (22). However, while liquid-culture-based type III secretion assays are generally reproducible, their throughput is limited due to the number of steps involved (see Fig. S1A in the supplemental material). To address this issue, we developed the first semiautomated solid-plate-based secretion assay.

10.1128/mBio.01050-18.1 FIG S1  *Shigella* type III effector secretion monitored by the liquid and the novel solid-plate-based secretion assays. (A) Schematic representation of the liquid secretion assay. Proteins were released by bacteria into CR-supplemented buffer, precipitated, separated via SDS-PAGE, and immunoblotted with antibodies of interest. (B) Immunoblots obtained from a solid-plate assay of WT *Shigella*. Sixty independent colonies were pinned, and overlaid membranes were incubated for 6 h, removed, and probed with anti-IpaD and anti-GroEL antibodies. Blots shown are representative of results from at least 3 independent experiments. CR, Congo red; LD, loading dye; OD, optical density; PBS, phosphate-buffered saline. Download FIG S1, PDF file, 0.2 MB. Copyright © 2018 Ernst et al. 2018 Ernst et al. This content is distributed under the terms of the Creative Commons Attribution 4.0 International license.

**FIG 1:**
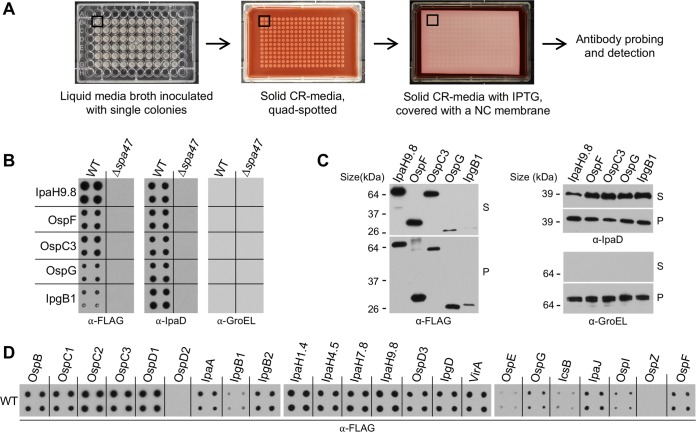
The solid-plate-based secretion assay reproducibly monitors *Shigella* type III effector secretion. (A) Schematic representation of the novel solid-plate-based secretion assay. Liquid cultures, grown in a 96-well format, are spotted in a quadruplicate (quad) manner onto solid CR-containing media using a pinning robot. After transfer of the colonies to a second plate, a nitrocellulose (NC) membrane is overlaid and the plate is then incubated at 37°C. Membranes are removed and probed with an antibody of interest. (B) Secretion of five designated IPTG-induced FLAG-tagged effectors by wild-type (WT) and Δ*spa47 Shigella* monitored via a 6-h solid-plate-based secretion assay. (C) Secretion of the same FLAG-tagged effectors by WT *Shigella* monitored via 30-min liquid secretion assays. With the exception of supernatant fractions derived from IpaH9.8, equal cell equivalents of whole-cell pellet lysates (P) and precipitated supernatant fractions (S) were analyzed. Twenty-five percent of the supernatant fraction of the more abundantly secreted IpaH9.8 was examined. (D) Secretion of 23 IPTG-driven FLAG epitope-tagged effectors by WT *Shigella* monitored via a 6-h solid-plate-based secretion assay. In each panel, all of the images shown are from the same exposure of three membranes immunoblotted with designated antibodies and are representative of results from at least 3 independent experiments. CR, Congo red.

The solid-plate-based assay (Fig. 1A) is performed with the assistance of a pinning robot. In the first step, the robot, outfitted with a 96-pin tool, is used for quadruplicate (quad) spotting of equivalent volumes of saturated liquid bacterial cultures onto CR-containing solid media. Following an overnight incubation, the robot, outfitted with a 384-pin tool, is used to transfer bacteria from the first tray onto a second CR-containing solid-medium tray, over which a nitrocellulose membrane is immediately laid. During a 6-to-18-h incubation at 37°C, released proteins are absorbed onto the membrane, which is subsequently removed, washed, and immunoblotted for the protein(s) of interest. Using this assay, we have observed similar amounts of IpaD, a component of the *Shigella* translocon, present within each of the four spots derived from a single culture, as well as between quad spots originating from separate independent cultures. In contrast, under the same conditions, we have observed no evidence of GroEL, a highly abundant cytoplasmic protein, demonstrating that the proteins deposited on the nitrocellulose membranes are not present due to bacterial lysis but rather are released from intact bacteria (Fig. S1B).

We next confirmed that the solid-plate assay monitors type III-dependent secretion and also examined the levels of effectors released via the solid and liquid secretion assays. For these studies, to directly compare the secretion of effectors, we studied the behavior of C-terminally 3×FLAG-tagged variants, each encoded downstream of a consensus Shine-Dalgarno sequence and expressed via an IPTG (isopropyl-β-d-thiogalactopyranoside)-inducible *lac* promoter (23, 24). Our initial studies focused on five FLAG-tagged effectors, each of which is released to nitrocellulose membranes by wild-type (WT) but not Δ*spa47 Shigella* (Fig. 1B), a strain that is secretion incompetent due to the absence of the essential T3SS ATPase (25). Notably, similar relative levels of the five effectors were observed to be secreted from WT *Shigella* via the solid-plate-based (Fig. 1B) and liquid secretion (Fig. 1C) assays, while GroEL was observed only in the pellet fractions of the liquid secretion assays, which contained intact bacterial cells (Fig. 1C). These observations confirm the validity of the solid-plate-based assay and demonstrate its functional complementarity with the conventional liquid assay.

We next investigated the ability of the solid secretion assay to detect the release of 23 different FLAG-tagged effectors from WT *Shigella* (Fig. 1D). After incubating the nitrocellulose membrane-overlain plate for 6 h, a time point that enables the detection of most effectors secreted at low levels without saturating the signals of those that are more robustly released, we observed secretion of all but two effectors. The secretion of one of these, OspZ, became detectable when a more sensitive chemiluminescence reagent was used (Fig. S2). Given our inability to detect secretion of OspD2, it was excluded from further studies. These observations provide the foundation for large-scale side-by-side comparative studies of the secretion levels of most *Shigella* effectors under different conditions.

10.1128/mBio.01050-18.2 FIG S2  Detection of OspZ secretion from *Shigella*. Secretion of IPTG-induced FLAG-tagged OspI, OspZ, and OspF from WT *Shigella* was monitored via a 6-h solid assay. The blot was probed with anti-FLAG antibody and exposed to different chemiluminescence reagents that detect picogram (pico) or femtogram (femto) protein amounts for indicated times. Blots shown are representative of results from at least 3 independent experiments. ECL, enhanced chemiluminescence. Download FIG S2, PDF file, 0.1 MB. Copyright © 2018 Ernst et al. 2018 Ernst et al. This content is distributed under the terms of the Creative Commons Attribution 4.0 International license.

### The majority of *Shigella* effectors are efficiently secreted independently of known T3S chaperones.

*Shigella* spp. encode three T3S class I effector chaperones. The class IA chaperones, IpgA and IpgE, are dedicated to the secretion of a single effector each, IcsB and IpgD, respectively (26, 27), whereas the class IB chaperone, Spa15, mediates the secretion of nine effectors, IpaA, IpgB1, IpgB2, OspB, OspC1, OspC2, OspC3, OspD1, and OspD2 (24, 28, 29) (Table 1). In prior systematic yeast two-hybrid (Y2H) and/or protein interaction platform assays, interactions were detected between each of these three chaperones and their respective 11 effectors (24). In contrast, with the exception of IpaH1.4, which interacted with Spa15, no interactions were detected between the three chaperones and 8 of the remaining 11 effectors listed in Table 1. (OspI and OspZ had not yet been discovered when these prior studies were conducted.)

**TABLE 1:** Summary of virulence plasmid-encoded *Shigella* effectors and their cognate chaperones

Effector	Chaperone (class)
IcsB	IpgA (IA)
IpgD	IpgE (IA)
IpaA	Spa15 (IB)
IpgB1	Spa15 (IB)
IpgB2	Spa15 (IB)
OspB	Spa15 (IB)
OspC1	Spa15 (IB)
OspC2	Spa15 (IB)
OspC3	Spa15 (IB)
OspD1	Spa15 (IB)
OspD2	Spa15 (IB)
IpaB	IpgC (II)
IpaC	IpgC (II)
IpaH1.4	
IpaH4.5	
IpaH7.8	
IpaH9.8	
IpaJ	
OspD3	
OspE	
OspF	
OspG	
OspI	
OspZ	
VirA	

**FIG 2:**
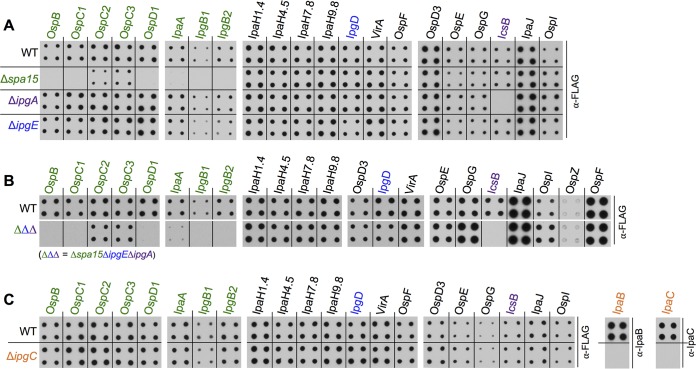
The majority of *Shigella* effectors are secreted independently of class I and II T3S chaperones. Six-hour solid-plate-based secretion assays were performed for analysis of each of the designated IPTG-induced FLAG-tagged effectors or translocon components, IpaB and IpaC, from wild type (WT) and *Shigella* deletion strains, each of which no longer encodes one class I chaperone (A) or all class I chaperones (IpgA, IpgE, and Spa15) (B) or the single *Shigella* class II chaperone (IpgC) (C). Nitrocellulose membranes were probed with indicated antibodies. The images shown in each panel are from a single assay of three solid-plate overlays, each treated in the same manner. In panels A and B, the images shown on the right are longer exposures than those of the left and middle panels, as they are images of effectors that are secreted at lower levels. Blots shown are representative of results from at least 3 independent experiments.

The observations summarized above suggested the existence of an as-yet-unknown chaperone(s) or the possibility that many *Shigella* effectors are secreted independently of known class I T3S chaperones. To investigate the latter, we directly compared the secretion levels of >20 FLAG-tagged *Shigella* effectors from WT, Δ*spa15*, Δ*ipgA*, and Δ*ipgE Shigella*, a feat that was not technically feasible prior to the development of our solid-plate-based secretion assay. After a 6-h incubation, as expected, we observed substantially decreased or absent secretion of each known chaperone-dependent effector from the deletion strain which lacks its cognate chaperone (Fig. 2A; see also Fig. S3A). Specifically, secretion of IcsB was not detected from Δ*ipgA Shigella*, while the secretion of each of the eight Spa15-dependent effectors from Δ*spa15 Shigella* was markedly impaired or absent. While the level of secretion of IpgD was decreased only modestly in the absence of its chaperone IpgE, we observed no evidence of IpgD secretion from Δ*ipgE Shigella* via a liquid secretion assay (Fig. S3B). As the liquid assay monitors secretion over 30 min and the solid-plate assay over 6 h, it appears that IpgE plays a key role in mediating early secretion of IpgD.

10.1128/mBio.01050-18.3 FIG S3  The majority of *Shigella* effectors are secreted independently of class I and II T3S chaperones. (A, C, and D) Six-hour solid-plate secretion assays of each of the designated IPTG-induced FLAG-tagged effectors or translocon components, IpaB and IpaC from wild-type (WT) and *Shigella* deletion strains, each of which no longer encodes one class I chaperone (A) or all class I chaperones (IpgA, IpgE, and Spa15) (C) or the single *Shigella* class II chaperone (IpgC) (D). Nitrocellulose membranes were probed with the indicated antibodies. The intensities of each quad were quantified using ImageJ. Heat maps were generated that depict the ratio of each protein secreted from the designated strain to the level of the same protein secreted from WT *Shigella*. The heat maps shown are representative of results from at least 3 independent experiments. (B) Secretion of IPTG-induced FLAG-tagged IpgD from WT, Δ*spa15*, Δ*ipgA* and Δ*ipgE Shigella* monitored via a 30-min liquid assay. Equal cell equivalents of proteins in the precipitated supernatant fractions (S) and whole-cell pellet lysates (P) were separated by SDS-PAGE and immunoblotted with anti-FLAG antibody. Blots shown are representative of results from at least 3 independent experiments. T3S, type III secretion. Download FIG S3, PDF file, 0.1 MB. Copyright © 2018 Ernst et al. 2018 Ernst et al. This content is distributed under the terms of the Creative Commons Attribution 4.0 International license.

The remaining 11 *Shigella* effectors are secreted at essentially the same levels from WT, Δ*spa15*, Δ*ipgA*, and Δ*ipgE Shigella* (Fig. 2A). Given the possibility that one or more class I chaperones might work in a functionally redundant or cooperative manner in mediating secretion, we wanted to test effector secretion from Δ*spa15ΔipgEΔipgA Shigella*, a strain that lacks all three class I T3S chaperones. Only the known chaperone-dependent effectors displayed markedly decreased or absent secretion from this strain (Fig. 2B; see also Fig. S3C), further supporting the assertion that most *Shigella* effectors are secreted independently of all currently known class I T3S chaperones.

Next, although the secretion of effectors has never been directly linked to a class II chaperone, we tested the secretion of *Shigella* effectors in the absence of its sole class II T3S chaperone, IpgC. As expected, the absence of IpgC had no effect on the secretion levels of effectors but resulted in decreased levels of secretion of the IpaB and IpaC translocon components (Fig. 2C; see also Fig. S3D), whose secretion is known to be dependent on IpgC (30). These findings demonstrate that the majority of *Shigella* effectors are efficiently secreted via a pathway independent of all known T3S chaperones.

### Chaperone-independent effectors are efficiently secreted by E. coli bacteria that express a functional *Shigella* T3SA.

**FIG 3:**
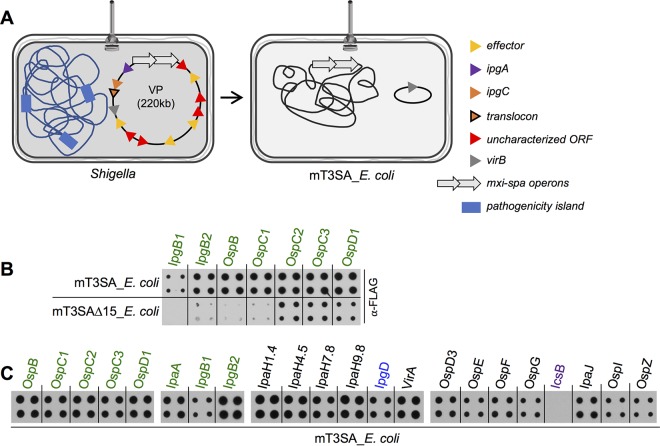
*Shigella* effectors are efficiently secreted by a core T3SA in E. coli. (A) Schematic representation of WT *Shigella* versus mT3SA_*E. coli.* The genetic elements transferred from *Shigella* into mT3SA_*E. coli* include VirB, a major T3SS transcriptional regulator, and the *mxi-spa* operons, which encode all the core structural components of the *Shigella* T3SA. (B) Secretion of designated Spa15-dependent IPTG-induced FLAG-tagged effectors from mT3SA_*E. coli* and mT3SAΔ*15*_*E. coli*. (C) Secretion of designated Spa15-dependent IPTG-induced FLAG-tagged effectors from 22 IPTG-induced FLAG-tagged effectors from mT3SA_*E. coli* monitored via a 6-h solid-plate assay. Nitrocellulose membranes were probed with anti-FLAG antibody. In panel C, the images shown are from the same exposure of three membranes. Blots shown are representative of results from at least 3 independent experiments. T3SA, type III secretion apparatus.

All of the proteins needed to form the *Shigella* T3SA, most of its effectors, and all of its T3S chaperones are encoded on a large ~220-kb virulence plasmid (VP) (31). Laboratory strains of E. coli that carry this plasmid invade and replicate within infected epithelial cells at even higher titers than WT *Shigella* (32), suggesting that it encodes all of the proteins involved in effector secretion. Additionally, the VP encodes >25 proteins of unknown function, one or more of which could potentially be a previously unidentified T3S chaperone. To investigate this possibility, we considered generating strains that no longer encode each of these proteins. However, given the possibility that two or more of these proteins might work in a functionally redundant manner, we used the following strategy to generate a means to study effector secretion in the absence of all proteins of unknown function. Using recombineering (33), we introduced the *mxi*-*spa* operons, which encode all structural components of the T3SA and two class I chaperones (IpgE and Spa15), into the chromosome of the nonpathogenic laboratory strain E. coli DH10β. The introduction of these operons, plus a plasmid that conditionally expresses the *mxi-spa* transcriptional regulator, VirB, resulted in the generation of mT3SA_*E. coli*, a type III secretion-competent strain, which contains only 8% of the *Shigella* virulence plasmid DNA and none of the chromosomally encoded *Shigella* pathogenicity islands (Fig. 3A).

mT3SA_*E. coli*, unlike WT *Shigella* and the chaperone deletion strains, encodes no effectors or translocon components. Thus, we first needed to establish that effector secretion remains chaperone dependent in the absence of competition for access to the T3SA. Thus, we compared the levels of Spa15-dependent effectors secreted by mT3SA_*E. coli* and mT3SAΔ*15*_*E. coli*, a strain that lacks the chaperone Spa15. Notably, the secretion of each was markedly diminished in the absence of Spa15 (Fig. 3B), establishing the relevancy of the use of this strain to study the secretory behavior of the putative chaperone-independent effectors. We next studied the secretion of our collection of FLAG-tagged *Shigella* effectors expressed in mT3SA_*E. coli* (Fig. 3C). As expected, we observed no evidence of IcsB secretion from mT3SA_*E. coli* due to the absence of its cognate chaperone IpgA. Strikingly, all the remaining effectors not only are efficiently secreted from mT3SA_*E. coli* but also are secreted at the same relative levels as were observed from WT *Shigella* (Fig. 1D) under the same experimental conditions. These observations demonstrate that none of the *Shigella-*specific proteins encoded outside the *mxi-spa* operons play a role in mediating effector secretion, thus strongly supporting the existence of a common chaperone-independent secretion pathway. However, they do not rule out the seemingly less likely existence of an as-yet-to-be-discovered new class of T3S chaperones, which would be the first shown not to be restricted to a single pathogen species but rather to be common to nonpathogenic E. coli and *Shigella*.

We next wanted to investigate whether any of the proteins present in mT3SA_*E. coli* might serve in the recruitment of chaperone-independent effectors to the machine. However, it was not possible to monitor effector secretion in their absence, as almost all of the proteins encoded within the well-studied *mxi-spa* operons (Table S2) are essential for secretion. Thus, to gain insights regarding how chaperone-independent effectors are recruited to the T3SA, we conducted an extensive yeast two-hybrid screen for binary interactions between effectors and cytoplasmic components of the T3SA. Specifically, we systematically tested for interactions between 17 effectors and 16 *mxi-spa*-encoded proteins. The latter included components of the sorting platform (MxiK, MxiN, Spa33, and Spa47), the export apparatus (MxiA, Spa9, Spa13, Spa24, Spa29, and Spa40), the basal body (MxiG), and regulators (Spa32, MxiC, MxiE, and MxiL) (31) and the multicargo chaperone Spa15. In the cases of MxiG and MxiA, we screened for interactions involving their predicted cytosolic domains (34, 35). No interactions, other than the previously observed interactions between Spa15 and its cognate effectors (24), were detected (Table S3), suggesting that cytosolically exposed T3SA proteins are not involved in the direct recruitment of either chaperone-dependent or chaperone-independent effectors to the T3SA.

10.1128/mBio.01050-18.6 TABLE S2  Proteins encoded in *mxi-spa* operons. Download TABLE S2, DOCX file, 0.02 MB. Copyright © 2018 Ernst et al. 2018 Ernst et al. This content is distributed under the terms of the Creative Commons Attribution 4.0 International license.

10.1128/mBio.01050-18.7 TABLE S3  Summary of Y2H studies. Download TABLE S3, DOCX file, 0.1 MB. Copyright © 2018 Ernst et al. 2018 Ernst et al. This content is distributed under the terms of the Creative Commons Attribution 4.0 International license.

### Chaperone-dependent and -independent effectors are defined by fundamentally different determinants.

**FIG 4:**
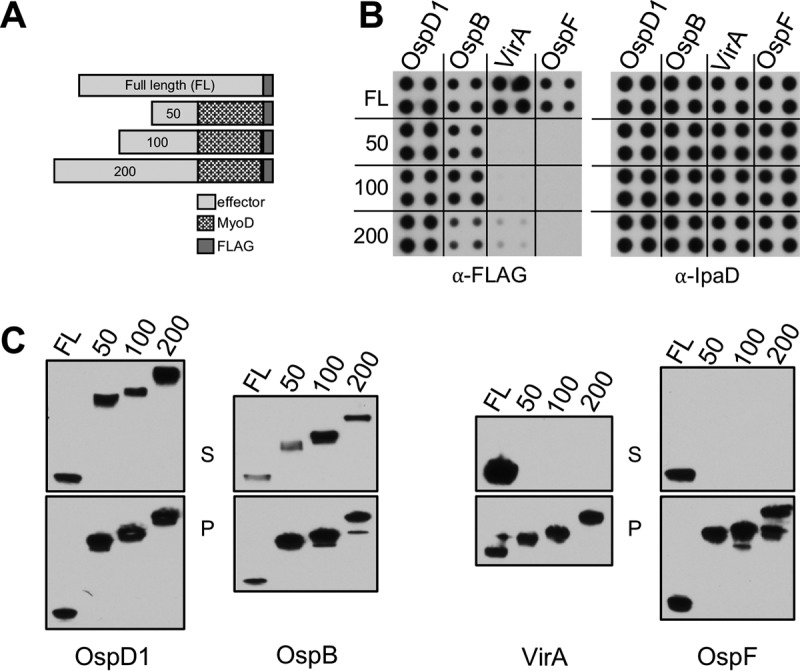
Secretion signals of chaperone-independent effectors are not limited to their amino termini. (A) Schematic representation of effector fusion proteins. (B and C) Secretion of designated IPTG-induced chaperone-dependent (OspD1 and OspB) and -independent (VirA and OspF) FLAG-tagged effector proteins from WT *Shigella* via a 6-h solid (B) or 30-min liquid (C) secretion assay. Equal cell equivalents of whole-cell pellet lysates (P) and precipitated supernatant fractions (S) were separated by SDS-PAGE and immunoblotted with anti-IpaD (B) or anti-FLAG (B and C) antibodies. Blots shown are representative of results from 3 independent experiments.

T3S effectors are commonly described as containing a bipartite secretion signal composed of an N-terminal secretion sequence followed by a downstream chaperone-binding domain (CBD). These effector domains have primarily been identified by studying the secretory behavior of heterologous proteins fused to N-terminal effector fragments (2, 36). As a first step toward comparing the sequences that define the chaperone-dependent and chaperone-independent effectors as secreted proteins, we studied the secretion of a heterologous mammalian protein that is normally not secreted, MyoD (37), fused to the first 50, 100, or 200 amino acids of two chaperone-dependent (OspD1 and OspB) and chaperone-independent (VirA and OspF) effectors (Fig. 4A). As expected, fusion of the first 50 residues of the two chaperone-dependent effectors, the sequences that contain their secretion sequences and previously mapped CBDs (38), is sufficient to generate secreted variants of MyoD-FLAG as assayed via either the solid-plate-based (Fig. 4B) or liquid (Fig. 4C) secretion assay. In fact, fusion to just the first 50 residues resulted in MyoD variants that were secreted at levels equivalent to those seen with each of the corresponding FLAG-tagged full-length effectors. In contrast, MyoD was not secreted when fused to the first 50, 100, or 200 amino acids of the tested chaperone-independent effectors, OspF and VirA, demonstrating that their N termini are not sufficient to mediate secretion. The absence of secretion was not due to T3SS inactivity, as equivalent levels of IpaD were secreted by each of the MyoD-expressing strains. It is also not due to instability of the fusion proteins, as they were present at roughly equivalent levels in the bacterial pellet fractions (Fig. 4C). Thus, the sequences of the chaperone-dependent and -independent effectors needed to mediate the recognition of MyoD as a type III secreted protein are substantially different.

**FIG 5:**
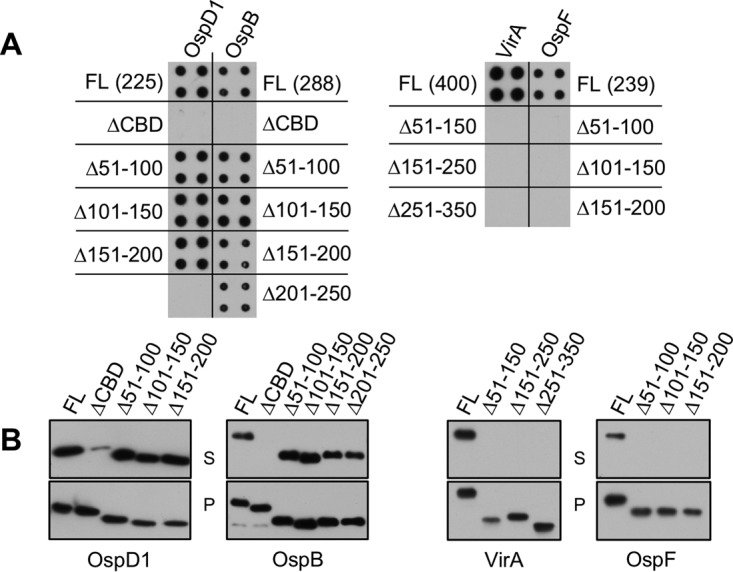
The sequences required for the secretion of chaperone-dependent and -independent effectors are fundamentally different. Secretion of designated deletion variants of chaperone-dependent (OspD1, OspB) and -independent (VirA, OspF) effectors was monitored via a 6-h solid-plate-based (A) or 30-min liquid (B) secretion assay. Equal cell equivalents of whole-cell pellet lysates (P) and precipitated supernatant fractions (S) were separated by SDS-PAGE and immunoblotted with anti-FLAG antibody. Blots shown are representative of results from at least 3 independent experiments. FL, full-length.

We next compared the sequences of the same chaperone-dependent (OspD1 and OspB) and -independent (VirA and OspF) effectors that are necessary for their secretion using a scanning deletion mutagenesis strategy. Depending on the size of the effector, we generated 50 to 100 amino acid deletions, smaller deletions for OspD1, OspB, and OspF and larger ones for VirA (Fig. S4A). In each case, we kept the first 50 residues intact, in order to not perturb potential N-terminal secretion sequences. In the case of the chaperone-dependent effectors, we also examined variants that no longer contained the 11 amino acids that corresponded to their previously mapped CBDs (38, 39). Via both the solid-plate-based (Fig. 5A) and liquid (Fig. 5B) secretion assays, we again observed fundamental differences in the sequences necessary to define chaperone-dependent and -independent effectors as secreted proteins. The only residues identified to play a role in defining chaperone-dependent effectors were those of the CBD, which is part of the bipartite secretion signal. In contrast, none of the mutated variants of the chaperone-independent effectors (VirA or OspF) were secreted despite being present at relatively equivalent levels in the total (Fig. 5B) and soluble (Fig. S4B) fractions of bacterial lysates. Furthermore, their lack of secretion was not due to inhibition of T3SS activity, as equivalent levels of secreted IpaD were observed under all conditions (Fig. S4C). Additional studies are needed to further refine the sequences essential for defining chaperone-independent effectors as secreted substrates. Nevertheless, these studies provided a clear demonstration that the sequences necessary and sufficient to define at least these chaperone-dependent and -independent effectors as secreted substrates are fundamentally different, thus providing further support for the existence of at least two distinct type III effector secretion pathways.

10.1128/mBio.01050-18.4 FIG S4  Expression of effector variants does not impact T3SA activity. (A) Schematic representation of chaperone-dependent (OspD1, OspB) and -independent (OspF, VirA) FLAG-tagged effector variants carrying designated deletions. (B) Immunoblots of sonicated fractions of WT *Shigella* expressing each of the designated effector variants. Equal cell equivalents of proteins in the pellet and supernatant fractions were separated by SDS-PAGE and immunoblotted with anti-FLAG and anti-DnaK antibodies. Blots shown are representative of results from at least 2 independent experiments. (C) Immunoblots of 6-h solid secretion assays of WT *Shigella* expressing each of the designated effector variants probed with anti-IpaD antibody. Blots shown are representative of results from at least 3 independent experiments. CBD, chaperone-binding domain; FL, full-length. Download FIG S4, PDF file, 0.4 MB. Copyright © 2018 Ernst et al. 2018 Ernst et al. This content is distributed under the terms of the Creative Commons Attribution 4.0 International license.

## DISCUSSION

In this report, we describe the development of a solid-plate-based secretion assay that enables, for the first time, the side-by-side concurrent analysis of secretion of >20 different *Shigella* effectors under multiple conditions. Remarkably, despite extensive evidence that effectors are both defined as secreted proteins (4, 6, 7) and recruited to the T3SA sorting platform via interactions with cognate chaperones (11), we found that the majority of *Shigella* effectors are efficiently secreted independently of all known T3S chaperones. Furthermore, we demonstrate that chaperone-independent effectors are efficiently secreted by mT3SA_*E. coli*, a laboratory strain of E. coli, which contains the operons needed to form a functional *Shigella* T3SA but none of the virulence-associated genes located within 92% of the *Shigella* virulence plasmid DNA nor within its chromosomal pathogenicity islands. This last observation suggests that if the secretion of chaperone-independent effectors is mediated via other, as-yet-unknown proteins, they are likely encoded in chromosomal regions conserved between *Shigella* and nonpathogenic E. coli DH10β and hence constitute a new class of T3S chaperones. Moreover, our observations strongly support the existence of two different modes of effector recognition, as the sequences that are necessary and sufficient to define chaperone-dependent and -independent effectors are fundamentally different. Thus, we propose that the chaperone-independent effectors are secreted via a previously unrecognized noncanonical secretion pathway.

A series of recent elegant imaging studies demonstrated that the membrane-embedded portion of the T3SA is static, exhibiting few structural changes between its resting and secreting states (40). In contrast, dynamic changes are observed at its cytosolic surface (9, 41, 42), where effectors are recruited and loaded into the export apparatus. These changes presumably reflect docking of the sorting platform, as it delivers effectors from the bacterial cytosol to the membrane-localized T3SA (8, 9, 34). These observations raise numerous questions regarding how chaperone-independent effectors are recruited to the T3SA. First, are they directly or indirectly recruited to the sorting platform? The latter seems more likely, as we observed no evidence of direct binding of effectors to components of the sorting platform via the Y2H assay. Similarly, other groups have not reported evidence of the chaperone-independent binding of effectors to the sorting platform (11). Alternatively, given previously reported observations that not all membrane-embedded T3SAs have associated sorting platforms (8, 9, 43), might chaperone-independent effectors be directly recruited by the export apparatus?

While our studies to systematically examine the roles of chaperones in mediating the secretion of a large complement of T3S effectors resulted in the discovery of widespread chaperone independence, a review of the literature suggests that chaperone independence is likely not an uncommon occurrence. For example, chaperones have been identified for only a third (38/109) of known effectors of the well-studied *Shigella*, *Salmonella*, *Yersinia*, or pathogenic E. coli T3SSs (12–19). In addition, as we observed for a few *Shigella* chaperone-dependent effectors, *Yersinia* YopE and YopH are inefficiently secreted in the absence of their cognate chaperones (38, 44), suggesting that under select conditions, even those effectors that bind chaperones might be secreted via a chaperone-independent pathway.

In summary, here, using a high-throughput semiautomated solid-plate-based *Shigella* secretion assay, we present evidence for the existence of a common noncanonical chaperone-independent type III secretion pathway. Future studies are needed to dissect the molecular details of this pathway. Nevertheless, given its prevalence, it offers a new and exciting target for the development of novel therapeutic agents in this emerging era of widespread antibiotic resistance.

## MATERIALS AND METHODS

Strains, plasmids, and oligonucleotides are summarized in Tables S4 and S5 in the supplemental material.

10.1128/mBio.01050-18.8 TABLE S4  Strain and plasmid summary. Download TABLE S4, DOCX file, 0.04 MB. Copyright © 2018 Ernst et al. 2018 Ernst et al. This content is distributed under the terms of the Creative Commons Attribution 4.0 International license.

10.1128/mBio.01050-18.9 TABLE S5  Oligonucleotide summary. Download TABLE S5, DOCX file, 0.01 MB. Copyright © 2018 Ernst et al. 2018 Ernst et al. This content is distributed under the terms of the Creative Commons Attribution 4.0 International license.

### Plasmid construction. (i) Effector-FLAG expression plasmids.

The *plac* (IPTG-inducible) effector 3×FLAG-tagged expression plasmids were generated as previously described (24, 45).

### (ii) Effector-MyoD-FLAG expression plasmids.

The first 150, 300, and 600 bp of the genes encoding OspD1, OspB, OspF, and VirA were PCR amplified from their corresponding pDSW206 expression plasmids using a 5′ oligonucleotide that binds to the vector upstream of the 5′ flanking *attB* site (DSW206 F) plus a gene/location-specific 3′ oligonucleotide, i.e., OspB_50 R. The amplified gene fragments were introduced into pDNR221 or pDNR223 via Gateway BP reactions to generate entry plasmids. After the gene fragment insertions were sequence verified, each was transferred into pDSW206-ccdB-MyoD-FLAG via a Gateway LR reaction.

### (iii) Effector deletion expression plasmids.

Each gene deletion was generated via splicing by overlap extension (SOEing) PCR using the following strategy. (i) Two first-round PCRs were conducted using the corresponding full-length gene-specific pDSW206 plasmid as a template. The upstream fragment was amplified using DSW206 F plus a gene/location-specific reverse oligonucleotide, i.e., OspB_51_100_3, while the downstream fragment was amplified using a gene/location-specific forward oligonucleotide, i.e., OspB_51_100_5, plus RrnB R, an oligonucleotide that binds downstream of the *attB* site. (ii) The two first-round fragments were then used as templates with Univ5 and RrnB R oligonucleotides to generate fragments that contained the desired deletions flanked by *attB* sites. The amplified fragments were introduced into pDNR221 via Gateway BP reactions. After the gene insertions were sequence verified, each was transferred into pDSW206-ccdB-FLAG via a Gateway LR reaction.

### (iv) Yeast expression plasmids.

Each yeast expression plasmid was generated via Gateway recombination, as previously described (24). Open reading frames (ORFs) encoding the proteins listed in Table S4 were PCR amplified in a closed (stop codon-containing) conformation. Those that contained Shine-Dalgarno sequences were generated using the seminested PCR strategy described above for OspI and OspZ. Those that did not were amplified via a single round of PCR. The amplified fragments were then introduced into either pDNR221 or pDNR223 to generate Gateway entry clones. In the case of OspF (K134A), a synthetic gBlock fragment (IDT, Skokie, IL) with flanking *attB* sites was introduced into pDNR223. After the sequences of the gene insertions were verified, effectors were introduced into pAD-ccdB, while components of the T3SA were introduced into pBD-ccdB via Gateway LR reactions.

### Strain construction. (i) *Shigella* deletion strains.

Each of the single-deletion strains (Table S4), except for Δ*ipgC Shigella*, was generated in S. flexneri 2457T via λ Red recombination (46) using the oligonucleotides described in Table S5. In each case, the kanamycin resistance (Kan^r^) cassette was resolved using FLP recombinase. The strain Δ*spa15 ΔipgE ΔipgA Shigella* was generated by first removing *ipgE* from Δ*spa15 Shigella* to generate Δ*spa15 ΔipgE*::KAN *Shigella*. After resolution of the Kan^r^ cassette, *ipgA* was then deleted from the strain to generate Δ*spa15 ΔipgE ΔipgA*::KAN *Shigella*.

### (ii) Generation of mT3SA_*E. coli* and mT3SAΔ*15*_*E. coli*.

mT3SA_*E. coli* was generated using a modified version of a previously described strategy (33, 37). First, a capture vector was generated that is designed to capture the region of virulence plasmid (VP) DNA present between the VirB promoter site located upstream of IpgD and Spa40. This was done by modifying pLLX13-*ipaJ-bla-spa40*, the original capture vector developed to capture the region of the virulence plasmid located between IpaJ and Spa40. Specifically, seminested PCR was used to generate the fragment of DNA present between *icsB* and *ipgD*. Gibson assembly was then used to swap this fragment with the original targeting sequence, sequence 1 of pLLX13-*ipaJ-bla-spa40*. After the integrity of this new capture vector, pLLX13-*icsB*/*ipgD-bla-spa40*, was confirmed via PCR, sequence analysis, and restriction digestion, it was transformed into E. coli DH10β, which carries a version of the *Shigella* virulence plasmid (VP *ΔipgD*::KAN), plus the λ Red recombinase. Homologous recombination was then used to introduce the desired region of VP DNA into the capture vector, thus generating pmT3SA. After the integrity of pmT3SA was confirmed by whole-plasmid sequencing, it was transformed into E. coli DH10β, which has a landing pad (LP) integration site at the *atp1*/*gidB* locus (47). The landing pad recombination system (48) was then used to introduce the region of captured DNA into the E. coli chromosome to generate mT3SAΔ*ipgD*::KAN_*E. coli*. The integrity of mT3SAΔ*ipgD*::KAN_*E. coli* was confirmed by PCR, after which the Kan^r^ cassette was removed to generate mT3SA_*E. coli.* The λ Red recombination system was then used to remove *spa15* to generate mT3SAΔ*spa15*::KAN*_E. coli.* The Kan^r^ cassette was resolved to generate mT3SAΔ*15*_*E. coli*.

### Liquid T3S assay.

Liquid secretion assays were performed as previously described (24). Overnight cultures grown in Trypticase soy (TCS) broth were diluted 1:100 into 2 ml of TCS broth and incubated for 100 min, at which time 1 mM IPTG was added to the cultures. After another 45 min of incubation, the optical density at 600 nm (OD_600_) of each bacterial culture was measured. Equivalent numbers of bacteria from each culture were pelleted, resuspended in 2 ml of phosphate-buffered saline (PBS)–10 µM Congo red (Sigma), and incubated for 30 min. All incubations were carried out at 37°C with aeration. Bacterial cultures were then centrifuged, and the cell pellets were resuspended in loading dye (40% glycerol, 240 mM Tris-HCl [pH 6.8], 8% SDS, 0.04% bromophenol blue, 5% beta-mercaptoethanol). After an additional centrifugation step was performed to remove the remaining intact bacteria, proteins in the supernatant fractions were precipitated using trichloroacetic acid (TCA) (10% [vol/vol]) and resuspended in loading dye. Equal cell equivalents of supernatant and pellet fractions were separated by sodium dodecyl sulfate-polyacrylamide gel electrophoresis (SDS-PAGE), transferred to nitrocellulose membranes, and immunoblotted with anti-FLAG (Sigma; F1804) (1:10,000), anti-IpaD (1:40,000) (Sigma), or anti-GroEL (Sigma; G6532) (1:100,000) antibodies. The anti-IpaD antibody was a generous gift from Wendy Picking, University of Kansas, Lawrence, KS.

### Solid-plate-based T3S assay.

A 96-well plate (Corning) containing TCS broth was inoculated with the designated strains and incubated with agitation for 6 to 18 h on a plate shaker. A BM3-BC pinning robot (S&P Robotics Inc., Toronto, Canada) outfitted with a 96-pin tool was then used to transfer equal volumes of saturated cultures onto solid trays (Nunc) that contained solid TCS media (Sigma) plus 10 µM Congo red (CR). Each colony was spotted in quadruplicate. After an overnight incubation, the BM3-BC pinning robot, outfitted with a 384-pin tool, was used to transfer bacteria to a solid-medium tray containing TCS media plus CR and 1 mM IPTG onto which a precut nitrocellulose membrane (Pierce) was immediately laid. All incubations were carried out at 37°C. After another 6 to 18 h of incubation, the overlaid membrane was removed, washed with buffer (Tris-buffered saline, 0.1% Tween 20) to eliminate attached cells, and then probed with one of the following antibodies: anti-FLAG or anti-IpaB (1:20,000), anti-IpaC (1:40,000), anti-IpaD, or anti-GroEL. The anti-IpaB, anti-IpaC, and anti-IpaD antibodies were generous gifts from Wendy Picking, University of Kansas, Lawrence, KS. Secretion was quantified using ImageJ (49), and secreted protein amounts relative to those seen with the WT strain were summarized in heat maps using Matrix3png (50).

### Solubility test.

Overnight cultures of WT S. flexneri 2457T grown in TCS broth were diluted 1:100 into 9 ml of TCS broth. After 100 min of incubation, 1 mM IPTG was added to the cultures. After another 45 min, on the basis of the OD_600_ readings, equivalent numbers of bacteria from each culture were pelleted. All incubations were carried out at 37°C with aeration. The bacterial pellets were resuspended in 2 ml of PBS containing protease inhibitor cocktail (Sigma) and sonicated on ice for 1 min for 2 cycles. The lysed cells were centrifuged, and equivalent cell volumes of supernatant and pellet fractions were separated by SDS-PAGE, transferred to nitrocellulose membranes, and immunoblotted with anti-FLAG and anti-DnaK (Abcam, Inc.; ab69617) (1:10,000).

### Y2H assay.

The Y2H pAD and pBD expression plasmids were introduced into MaV103 and MaV203, respectively. The Y2H assays were performed in a 96-well format as previously described (24, 51). In this case, selection was conducted on medium that lacked leucine, tryptophan, and histidine and that included 30 to 50 mM 3-amino-1,2,4-triazole. Growth was scored after 3 days of incubation at 30°C.
