# Supplementary material for: High-Throughput Screening of Type III Secretion Determinants Reveals a Major Chaperone-Independent Pathway
Source: mBio. 2018 Jun 19;9(3):e01050-18. doi: 10.1128/mBio.01050-18 (PMC6016238; doi:10.1128/mBio.01050-18)
Supplement: TABLE S1 [file mbo003183931st1.docx]

| **Table S1: Summary of effectors and their cognate chaperones** | | |
| --- | --- | --- |
| **Effector** | **Chaperone** | **Class** |
| ***Shigella* (Mxi-Spa T3SS)** |  |  |
| **Virulence-plasmid-encoded** |  |  |
| IpaA | Spa15 | IB |
| IpgB1 | Spa15 | IB |
| IpgB2 | Spa15 | IB |
| OspB | Spa15 | IB |
| OspC1 | Spa15 | IB |
| OspC2 | Spa15 | IB |
| OspC3 | Spa15 | IB |
| OspD1 | Spa15 | IB |
| OspD2 | Spa15 | IB |
| IcsB | IpgA | IA |
| IpgD | IpgE | IA |
| IpaH1.4 | - | - |
| IpaH4.5 | - | - |
| IpaH7.8 | - | - |
| IpaH9.8 | - | - |
| IpaJ | - | - |
| OspD3 | - | - |
| OspE1 | - | - |
| OspE2 | - | - |
| OspF | - | - |
| OspG | - | - |
| OspI | - | - |
| OspZ | - | - |
| VirA | - | - |
| **Chromosomally-encoded** |  |  |
| IpaH_1 | - | - |
| IpaH_2 | - | - |
| IpaH_3 | - | - |
| IpaH_4 | - | - |
| IpaH_5 | - | - |
| IpaH_6 | - | - |
| IpaH_7 | - | - |
|  |  |  |
| ***Yersinia* (Ysc T3SS)** |  |  |
| YopE | SycE | IA |
| YopJ | - | - |
| YopH | sycH | IA |
| YopM | - | - |
| YopT | SycT | IA |
| YpkA/YopO | SycO | IA |
|  |  |  |
| ***Salmonella* (SPI1 T3SS)** |  |  |
| SipA (SspA) | InvB | IB |
| SopA | InvB | IB |
| SopE2 | InvB | IB |
| SopE1 | InvB | IB |
| SopB | SigE | IA |
| AvrA | - | - |
| SopD | - | - |
| SptP | SicP | IA |
| SspH1 | - | - |
| SpvC | - | - |
| SlrP | - | - |
| ***Salmonella* (SPI2 T3SS)** |  |  |
| PipB | - | - |
| PipB2 | SrcA | IB |
| SseL | SrcA | IB |
| SopD2 | - | - |
| SifA | - | - |
| SseJ | - | - |
| SseI | - | - |
| SspH2 | - | - |
| SpvB | - | - |
| SseF | SscB | IA? |
| SseG | SscB? | ? |
| SteC | - | - |
| Slrp1 | - | - |
| GogB | - | - |
| SifB | - | - |
| SpiC | - | - |
| SseK1 | - | - |
| SseK2 | - | - |
| SteA | - | - |
| SteB | - | - |
|  |  |  |
| ***Escherichia* *coli* (Esc T3SS)** |  |  |
| Tir | CesT | IB |
| Map | CesT | IB |
| EspH | CesT | IB |
| EspJ | CesT | IB |
| EspZ | CesT | IB |
| NleA/EspI | CesT | IB |
| NleB1 | CesT | IB |
| NleB2 | CesT | IB |
| NleC | CesT | IB |
| NleG | CesT | IB |
| NleH1 | CesT | IB |
| NleH2 | CesT | IB |
| EspF | CesF | IA |
| EspFU | - | - |
| EspG | - | - |
| EspG2 | - | - |
| EspK | - | - |
| EspL | - | - |
| EspL2 | - | - |
| EspM | - | - |
| EspN | - | - |
| EspO | - | - |
| EspR | - | - |
| EspS | - | - |
| EspT | - | - |
| EspV | - | - |
| EspW | - | - |
| EspX | - | - |
| EspY | - | - |
| NleB3 | - | - |
| NleD | - | - |
| NleE | - | - |
| NleE2 | - | - |
| NleF | - | - |
| NleH3 | - | - |
| NleI | - | - |
| NleJ | - | - |
| NleK | - | - |
| NleL (ESPX7) | - | - |
| Cif | - | - |
| Efa1 | - | - |
