# Supplementary figures and images for: High-Throughput Screening of Type III Secretion Determinants Reveals a Major Chaperone-Independent Pathway
Source: mBio. 2018 Jun 19;9(3):e01050-18. doi: 10.1128/mBio.01050-18 (PMC6016238; doi:10.1128/mBio.01050-18)

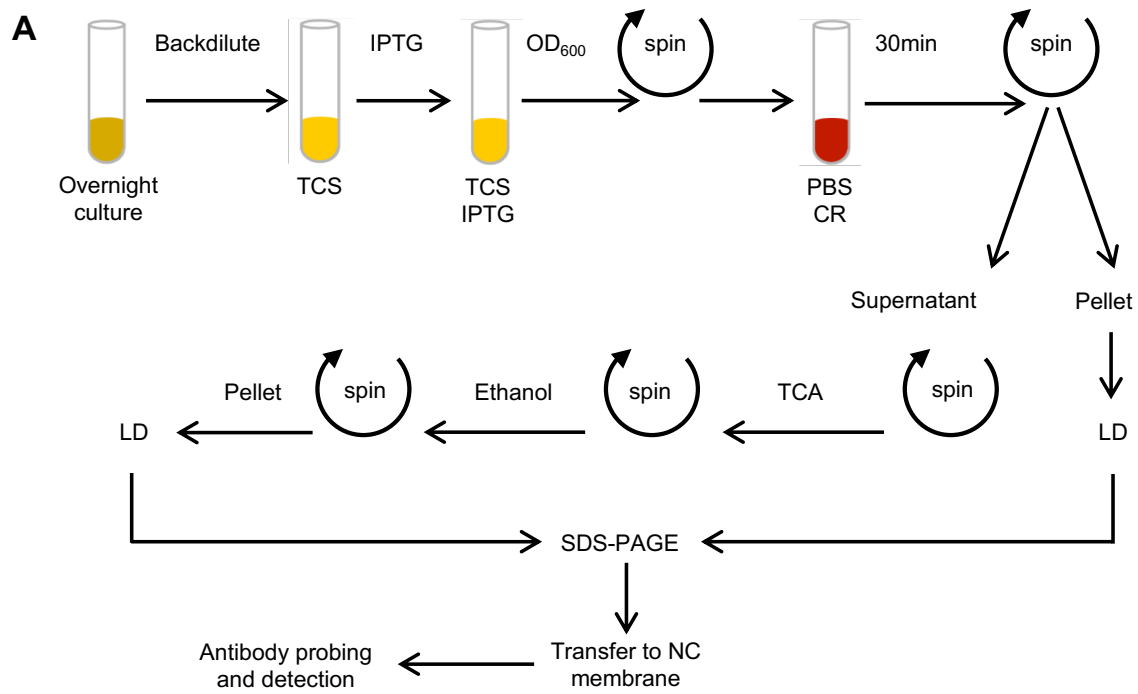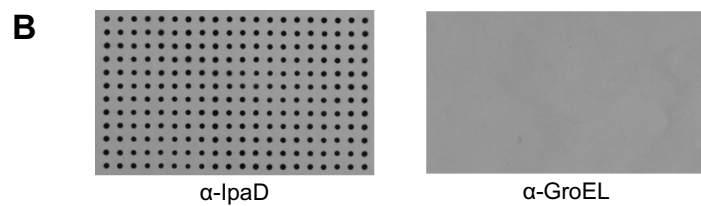

Supplement: FIG S1 [file mbo003183931sf1.pdf]

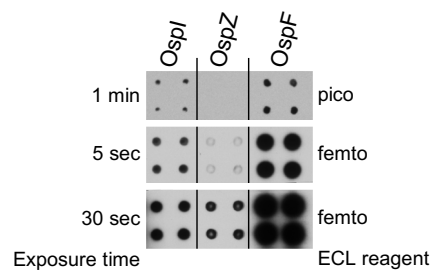

Supplement: FIG S2 [file mbo003183931sf2.pdf]

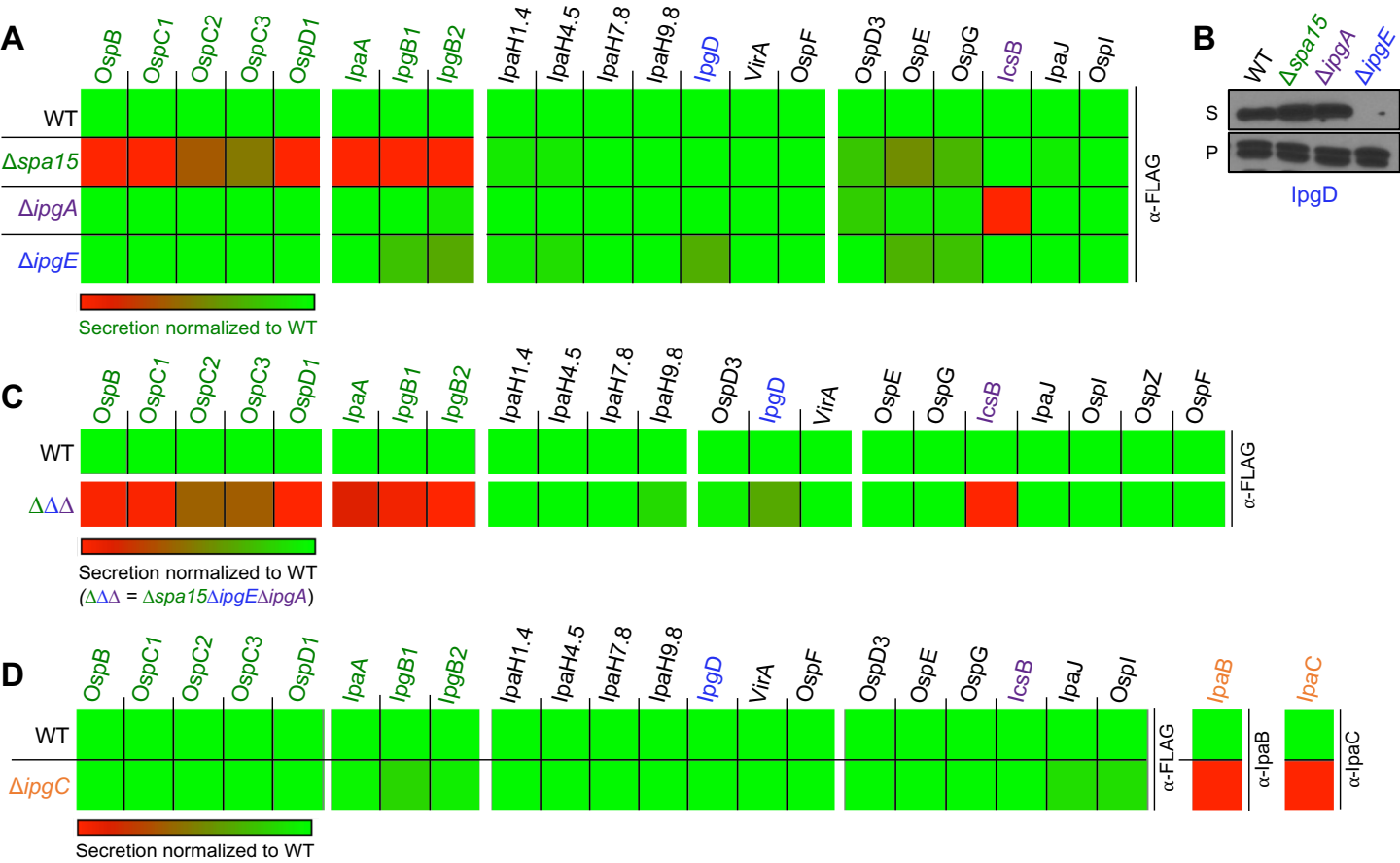

Supplement: FIG S3 [file mbo003183931sf3.pdf]

**A**

Chaperone-dependent effectors:

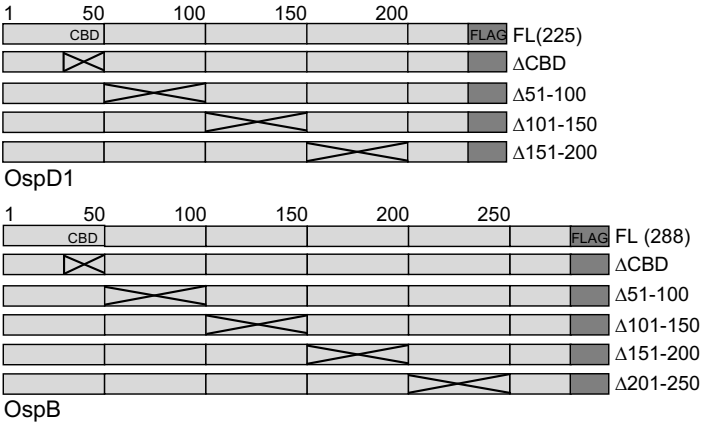

Chaperone-independent effectors:

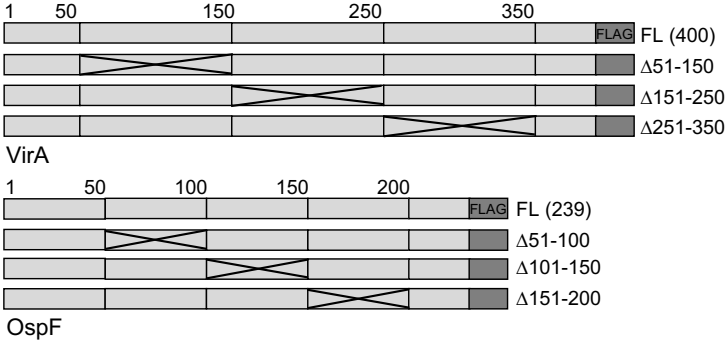

**B**

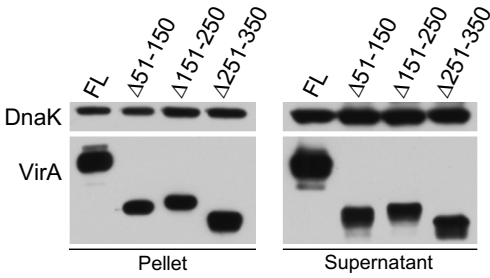

**C**

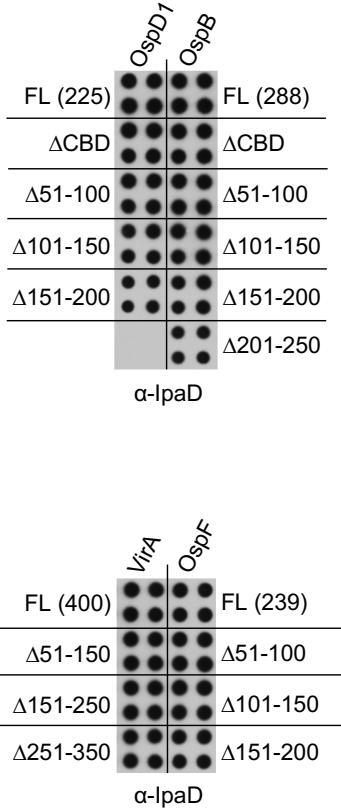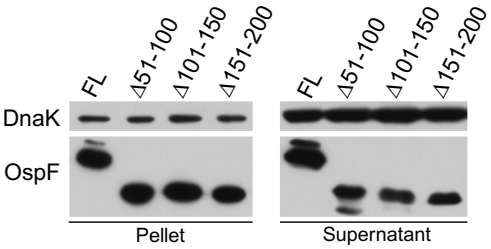

Supplement: FIG S4 [file mbo003183931sf4.pdf]
