# Supplementary material for: High-Throughput Screening of Type III Secretion Determinants Reveals a Major Chaperone-Independent Pathway
Source: mBio. 2018 Jun 19;9(3):e01050-18. doi: 10.1128/mBio.01050-18 (PMC6016238; doi:10.1128/mBio.01050-18)
Supplement: TABLE S2 [file mbo003183931st2.docx]

| **Table S2**. **Proteins encoded in *mxi-spa* operons.** | | |
| --- | --- | --- |
| **Protein** | **Function** (1-3) | **Location** (3,4) |
| Spa15 | class I chaperone | cytoplasm |
| IpgE | class I chaperone | cytoplasm |
| MxiE | transcription factor | cytoplasm |
| MxiK | sorting platform | cytoplasm |
| MxiN | sorting platform | cytoplasm |
| Spa33 | sorting platform | cytoplasm |
| Spa47 | ATPase | cytoplasm |
| Spa13 | ATPase-associated stalk | cytoplasm |
| MxiC | gatekeeper | cytoplasm |
| Spa9 | export apparatus | cytoplasmic membrane |
| Spa24 | export apparatus | cytoplasmic membrane |
| Spa29 | export apparatus | cytoplasmic membrane |
| Spa40 | export apparatus | cytoplasmic membrane |
| MxiA | export apparatus | cytoplasmic membrane with large cytoplasmic domain |
| MxiG | needle complex inner rings | cytoplasmic membrane with large cytoplasmic domain |
| MxiJ | needle complex inner rings | cytoplasmic membrane |
| MxiI | inner rod | inside of basal body |
| MxiD | needle complex outer rings | outer membrane |
| MxiH | needle filament | external |
| Spa32 | ruler protein, needle-length control | inside of basal body |
| IpgF | peptidoglycase | n/a |
| MxiM | pilotin, assembly of outer rings | outer membrane |
| MxiL | postulated regulator, secreted | n/a |

**References**

1. Galan, J. E., Lara-Tejero, M., Marlovits, T. C., and Wagner, S. (2014) Bacterial type III secretion systems: specialized nanomachines for protein delivery into target cells. *Annu Rev Microbiol* **68**, 415-438

2. Cherradi, Y., Hachani, A., and Allaoui, A. (2014) Spa13 of *Shigella flexneri* has a dual role: chaperone escort and export gate-activator switch of the type III secretion system. *Microbiology* **160**, 130-141

3. Zahrl, D., Wagner, M., Bischof, K., Bayer, M., Zavecz, B., Beranek, A., Ruckenstuhl, C., Zarfel, G. E., and Koraimann, G. (2005) Peptidoglycan degradation by specialized lytic transglycosylases associated with type III and type IV secretion systems. *Microbiology* **151**, 3455-3467

4. Hu, B., Morado, D. R., Margolin, W., Rohde, J. R., Arizmendi, O., Picking, W. L., Picking, W. D., and Liu, J. (2015) Visualization of the type III secretion sorting platform of *Shigella flexneri*. *Proc Natl Acad Sci U S A* **112**, 1047-1052
