# Supplementary material for: High-Throughput Screening of Type III Secretion Determinants Reveals a Major Chaperone-Independent Pathway
Source: mBio. 2018 Jun 19;9(3):e01050-18. doi: 10.1128/mBio.01050-18 (PMC6016238; doi:10.1128/mBio.01050-18)
Supplement: TABLE S4 [file mbo003183931st4.docx]

| **Table S4. Strain and plasmid summary.** | | |
| --- | --- | --- |
| **Strain/Plasmid** | **Characteristics** | **Source** |
| Strain: |  |  |
| *Shigella flexneri* 2457T serotype 2a | wildtype | (1) |
| ∆*ipgA::*KAN *Shigella* | ∆*ipgA*::FRT-KAN^R^-FRT | This study |
| ∆*ipgA Shigella* | ∆*ipgA*::FRT | This study |
| ∆*ipgE::*KAN *Shigella* | ∆*ipgE*::FRT-KAN^R^-FRT | This study |
| ∆*ipgE Shigella* | ∆*ipgE*::FRT | This study |
| ∆*spa15 Shigella* | ∆*spa15*::FRT | (2) |
| ∆*spa15*∆*ipgE::*KAN *Shigella* | ∆*spa15*∆*ipgE*::FRT-KAN^R^-FRT | This study |
| ∆*spa15*∆*ipgE Shigella* | ∆*spa15*∆*ipgE*::FRT |  |
| ∆*spa15*∆*ipgE*∆*ipgA::*KAN *Shigella* | ∆*spa15*∆*ipgE*∆*ipgA*::FRT-KAN^R^-FRT | This study |
| ∆*spa47::*KAN *Shigella* | ∆*spa47*::FRT-KAN^R^-FRT | This study |
| ∆*spa47 Shigella* | ∆*spa47*::FRT | This study |
| *Shigella flexneri* M90T serotype 5a | wildtype | (3) |
| ∆*ipgC::*TET *Shigella* M90T | ∆*ipgC*::FRT-TET^R^-FRT | (4) |
| *Escherichia coli* DH10β |  | Invitrogen |
| *E.coli* DH10β *atp/gidB::*LP | *atp/gidB::*Landing Pad cassette, TET^R^ | Addgene (83036) |
| mT3SA∆*ipgD::*KAN_*E.coli* | mT3SA (intergenic region of icsB and IpgD*∆ipgD*::FRT-KAN^R^-FRT -*ipgE* thru *spa40*, ~18kb) integrated in chromosome | This study |
| mT3SA_*E.coli* | mT3SA (intergenic region of icsB and IpgD∆ipgD::FRT-*ipgE* thru *spa40*, ~18kb) integrated in chromosome | This study |
| mT3SA∆*15::*KAN_*E.coli* | mT3SA (intergenic region of icsB and IpgD∆ipgD::FRT-*ipgE* thru *mxiA∆spa15*::FRT-KAN^R^-FRT thru *spa40*) integrated in chromosome | This study |
| mT3SA∆*15*_*E.coli* | mT3SA (intergenic region of icsB and IpgD∆ipgD::FRT-*ipgE* thru *mxiA∆spa15*::FRT thru *spa40*) integrated in chromosome | This study |
| Plasmid: |  |  |
| pCP20 | Temperature sensitive, AMP^R^, FLP recombinase | (5) |
| pKD46 | Temperature sensitive, λ Red recombinase, AMP^R^ | (5) |
| pLLX13-ipaJ-bla-spa40 | T3SS capture vector, AMP^R^, Tet^R^, incP ori | (6) |
| pLLX13-icsB/ipgD-bla-spa40 | T3SS capture vector to generate pmT3SA, AMP^R^, TET^R^ | This study |
| pmT3SA | pLLX13 that carries T3SA genes from pVP ∆*ipgD*::FRT-KAN^R^-FRT (*icsB*/*ipgD*∆*ipgD*::FRT-Kan^R^-FRT thru *spa40*), incP ori, TET^R^, KAN^R^ | This study |
| pTKRED | Temperature sensitive, λ Red recombinase and I-SceI endonuclease, SPEC^R^ | (7) |
| ∆*ipgD*::KAN VP | ∆*ipgD*::FRT-KAN^R^-FRT virulence plasmid from *S. flexneri* 2457T carrying deletion of *ipgD* | (8) |
| **Entry plasmids** | | |
| pDNR221 | Gateway^®^ donor vector, pUC ori, KAN^R^ | Invitrogen |
| pENTR221-ospB(50) | Entry clone, 1-150bp of *ospB*, open configuration | This study |
| pENTR221-ospB(100) | Entry clone, 1- 300bp of *ospB*, open configuration | This study |
| pENTR221-ospB(200) | Entry clone, 1-600bp of *ospB*, open configuration | This study |
| pENTR221-ospB∆51-100 | Entry clone, with 151-300bp deletion in *ospB*, open configuration | This study |
| pENTR221-ospB∆101-150 | Entry clone, with 301-450bp deletion in *ospB*, open configuration | This study |
| pENTR221-ospB∆151-200 | Entry clone, with 451-600bp deletion in *ospB*, open configuration | This study |
| pENTR221-ospB∆201-250 | Entry clone, with 601-750bp deletion in *ospB*, open configuration | This study |
| pENTR221-ospD1(50) | Entry clone, 1-150bp of *ospD1*, open configuration | This study |
| pENTR221-ospD1(100) | Entry clone, 1- 300bp of *ospD1*, open configuration | This study |
| pENTR223-ospD1(200) | Entry clone, 1-600bp of *ospD1*, open configuration | This study |
| pENTR221-ospD1∆51-100 | Entry clone, with 151-300bp deletion in *ospD1*, open configuration | This study |
| pENTR221-ospD1∆101-150 | Entry clone, with 301-450bp deletion in *ospD1*, open configuration | This study |
| pENTR221-ospD1∆151-200 | Entry clone, with 451-600bp deletion in *ospD1*, open configuration | This study |
| pENTR221-ospF(50) | Entry clone, 1-150bp of *ospF*, open configuration | This study |
| pENTR221-ospF(100) | Entry clone, 1- 300bp of *ospF*, open configuration | This study |
| pENTR221-ospF(200) | Entry clone, 1-600bp of *ospF*, open configuration | This study |
| pENTR221-ospF∆51-100 | Entry clone, with 151-300bp deletion in *ospF*, open configuration | This study |
| pENTR221-ospF∆101-150 | Entry clone, with 301-450bp deletion in *ospF*, open configuration | This study |
| pENTR221-ospF∆151-200 | Entry clone, with 451-600bp deletion in *ospF*, open configuration | This study |
| pENTR221-ospI | Entry clone, open and closed configuration | (9) |
| pENTR221-ospZ | Entry clone, open and closed configuration | (9) |
| pENTR221-mxiL | Entry clone, closed configuration | This study |
| pENTR221-spa33 | Entry clone, yeast codon optimized, closed configuration | This study |
| pENTR221-virA(50) | Entry clone, 1-150bp of *virA*, open configuration | This study |
| pENTR221-virA∆51-150 | Entry clone, with 151-450bp deletion in *virA*, open configuration | This study |
| pENTR221-virA∆151-250 | Entry clone, with 451-750bp deletion in *virA*, open configuration | This study |
| pENTR221-virA∆251-350 | Entry clone, with 751-1050bp deletion in *virA*, open configuration | This study |
| pDNR223 | Gateway donor vector, pUC ori, SPEC^R^ | Invitrogen |
| pENTR223-ipgF | Entry clone, closed configuration | This study |
| pDNR223-mxiA(318-386) | Entry clone, 952-2061 bp of *mxiA*, closed configuration | This study |
| pENTR223-mxiC | Entry clone, closed configuration | This study |
| pENTR223-mxiE | Entry clone, closed configuration | This study |
| pENTR223-mxiG(1-126) | Entry clone, 1-378 bp of *mxiG*, closed configuration | This study |
| pENTR223-mxiK | Entry clone, closed configuration | This study |
| pENTR223-mxiN | Entry clone, closed configuration | This study |
| pENTR223-ospF(K134A) | Entry clone, OspF K134A, closed configuration | This study |
| pENTR223-spa9 | Entry clone, closed configuration | This study |
| pENTR223-spa13 | Entry clone, closed configuration | This study |
| pENTR223-spa24 | Entry clone, closed configuration | This study |
| pENTR223-spa29 | Entry clone, closed configuration | This study |
| pENTR223-spa32 | Entry clone, closed configuration | This study |
| pENTR223-spa40 | Entry clone, closed configuration | This study |
| pENTR223-spa47 | Entry clone, closed configuration | This study |
| pENTR223-virA(100) | Entry clone, 1- 300bp of *virA*, open configuration | This study |
| pENTR223-virA(200) | Entry clone, 1-600bp of *virA*, open configuration | This study |
| **Effector-FLAG expression plasmids** | | |
| pDSW206-ccdB-FLAG | Destination vector, 3xFLAG, p*lac* (IPTG), low copy (ColE1 ori), AMP^R^ | (2) |
| pDSW206-IcsB-FLAG | Expression clone, effector ORF, 3xFLAG, p*lac* (IPTG), low copy (ColE1 ori), AMP^R^ | (2) |
| pDSW206-ipaA-FLAG | “ | (2) |
| pDSW206-ipaJ-FLAG | “ | (2) |
| pDSW206-ipaH1.4-FLAG | “ | (2) |
| pDSW206-ipaH4.5-FLAG | “ | (2) |
| pDSW206-ipaH7.8-FLAG | “ | (2) |
| pDSW206-ipaH9.8-FLAG | “ | (2) |
| pDSW206-ipgB1-FLAG | “ | (2) |
| pDSW206-ipgB2-FLAG | “ | (2) |
| pDSW206-ipgD-FLAG | “ | (2) |
| pDSW206-ospB-FLAG | “ | (2) |
| pDSW206-ospC1-FLAG | “ | (2) |
| pDSW206-ospC2-FLAG | “ | (2) |
| pDSW206-ospC3-FLAG | “ | (2) |
| pDSW206-ospD1-FLAG | “ | (2) |
| pDSW206-ospD2-FLAG | “ | (2) |
| pDSW206-ospD3-FLAG | “ | (2) |
| pDSW206-ospE-FLAG | “ | (2) |
| pDSW206-ospF-FLAG | “ | (2) |
| pDSW206-ospG-FLAG | “ | (2) |
| pDSW206-ospI-FLAG | “ | (9) |
| pDSW206-ospZ-FLAG | “ | (9) |
| pDSW206-virA-FLAG | “ | (2) |
| pNG162-virB | Expression clone, p*lac* (IPTG), *virB*, low copy (SC101 ori), SPEC^R^ | (6) |
| **Effector-MyoD-FLAG expression plasmids** | | |
| pDSW206-ccdB-myoD-FLAG | Destination vector, p*lac* (IPTG), *myoD*-3xFLAG, low copy (ColE1 ori), AMP^R^ |  |
| pDSW206-ospB(50)-myoD-FLAG | expression clone, *effector*(bp)-*myoD*-3xFLAG, low copy (ColE1 ori), AMP^R^ | This study |
| pDSW206-ospB(100)-myoD-FLAG | “ | This study |
| pDSW206-ospB(200)-myoD-FLAG | “ | This study |
| pDSW206-ospD1(50)-myoD-FLAG | “ | This study |
| pDSW206-ospD1(100)-myoD-FLAG | “ | This study |
| pDSW206-ospD1(200)-myoD-FLAG | “ | This study |
| pDSW206-ospF(50)-myoD-FLAG | “ | This study |
| pDSW206-ospF(100)-myoD-FLAG | “ | This study |
| pDSW206-ospF(200)-myoD-FLAG | “ | This study |
| pDSW206-virA(50)-myoD-FLAG | “ | This study |
| pDSW206-virA(100)-myoD-FLAG | “ | This study |
| pDSW206-virA(200)-myoD-FLAG | “ | This study |
| **Effector deletion expression plasmids** | | |
| pDSW206-ospB∆cbd-FLAG | Expression clone, *effector*∆deletion-3xFLAG, p*lac* (IPTG), low copy (ColE1 ori), AMP^R^ | (10) |
| pDSW206-ospB∆51-100-FLAG | “ | This study |
| pDSW206-ospB∆101-150-FLAG | “ | This study |
| pDSW206-ospB∆151-200-FLAG | “ | This study |
| pDSW206-ospD1∆cbd-FLAG | “ | (10) |
| pDSW206-ospD1∆51-100-FLAG | “ | This study |
| pDSW206-ospD1∆101-150-FLAG | “ | This study |
| pDSW206-ospD1∆151-200-FLAG | “ | This study |
| pDSW206-ospF∆51-100-FLAG | “ | This study |
| pDSW206-ospF∆101-150-FLAG | “ | This study |
| pDSW206-ospF∆151-200-FLAG | “ | This study |
| pDSW206-virA∆51-150-FLAG | “ | This study |
| pDSW206-virA∆151-250-FLAG | “ | This study |
| pDSW206-virA∆251-350-FLAG | “ | This study |
| **Y2H expression plasmids** | | |
| pAD-ccdB | Destination vector, GAL4AD-fusion, low copy (cen), LEU, AMP^R^ | (11) |
| pAD-ipaA | Expression vector, GAL4AD-fusion, low copy (cen), LEU, AMP^R^ | (2) |
| pAD-ipaH1.4 | “ | (2) |
| pAD-ipaH4.5 | “ | (2) |
| pAD-ipaH7.8 | “ | (2) |
| pAD-ipaH9.8 | “ | (2) |
| pAD-ipgB1 | “ | (2) |
| pAD-ospB | “ | (2) |
| pAD-ospC1 | “ | (2) |
| pAD-ospC2 | “ | (2) |
| pAD-ospC3 | “ | (2) |
| pAD-ospD1 | “ | (2) |
| pAD-ospD2 | “ | (2) |
| pAD-ospD3 | “ | (2) |
| pAD-ospE | “ | (2) |
| pAD-ospF(K134A) | “ | This study |
| pAD-ospG | “ | (2) |
| pAD-ospZ | “ | This study |
| pBD-ccdB | Destination vector, GAL4BD-fusion, low copy (cen), TRP, KAN^R^ | (11) |
| pBD-ipgF | Expression vector, GAL4BD-fusion, low copy (cen), TRP, KAN^R^ | This study |
| pBD-mxiA(318-386) | “ | This study |
| pBD-mxiC | “ | This study |
| pBD-mxiE | “ | This study |
| pBD-mxiG(1-126) | “ | This study |
| pBD-mxiK | “ | This study |
| pBD-mxiL | “ | This study |
| pBD-mxiN | “ | This study |
| pBD-spa9 | “ | This study |
| pBD-spa13 | “ | This study |
| pBD-spa15 | “ | (8) |
| pBD-spa24 | “ | This study |
| pBD-spa29 | “ | This study |
| pBD-spa32 | “ | This study |
| pBD-spa33 | “ | This study |
| pBD-spa40 | “ | This study |
| pBD-spa47 | “ | This study |

**References**

1. Labrec, E. H., Schneider, H., Magnani, T. J., and Formal, S. B. (1964) Epithelial Cell Penetration as an Essential Step in the Pathogenesis of Bacillary Dysentery. *J Bacteriol* **88**, 1503-1518

2. Schmitz, A. M., Morrison, M. F., Agunwamba, A. O., Nibert, M. L., and Lesser, C. F. (2009) Protein interaction platforms: visualization of interacting proteins in yeast. *Nat Methods* **6**, 500-502

3. Onodera, N. T., Ryu, J., Durbic, T., Nislow, C., Archibald, J. M., and Rohde, J. R. (2012) Genome sequence of *Shigella* *flexneri* serotype 5a strain M90T Sm. *J Bacteriol* **194**, 3022

4. Sidik, S., Kottwitz, H., Benjamin, J., Ryu, J., Jarrar, A., Garduno, R., and Rohde, J. R. (2014) A *Shigella* *flexneri* virulence plasmid encoded factor controls production of outer membrane vesicles. *G3 (Bethesda)* **4**, 2493-2503

5. Datsenko, K. A., and Wanner, B. L. (2000) One-step inactivation of chromosomal genes in *Escherichia* *coli* K-12 using PCR products. *Proc Natl Acad Sci U S A* **97**, 6640-6645

6. Reeves, A. Z., Spears, W. E., Du, J., Tan, K. Y., Wagers, A. J., and Lesser, C. F. (2015) Engineering *Escherichia* *coli* into a protein delivery system for mammalian cells. *ACS Synth Biol* **4**, 644-654

7. Kuhlman, T. E., and Cox, E. C. (2010) Site-specific chromosomal integration of large synthetic constructs. *Nucleic Acids Res* **38**, e92

8. Du, J., Reeves, A. Z., Klein, J. A., Twedt, D. J., Knodler, L. A., and Lesser, C. F. (2016) The type III secretion system apparatus determines the intracellular niche of bacterial pathogens. *Proc Natl Acad Sci U S A* **113**, 4794-4799

9. Mou, X. S., S; Du, J; Reeves, AZ; Lesser, CF. (2018, in press) A novel synthetic bottom-up approach reveals the complex interplay of *Shigella* effectors in regulation of epithelial cell death. . *Proc Natl Acad Sci U S A*

10. Costa, S. C., and Lesser, C. F. (2014) A multifunctional region of the *Shigella* type 3 effector IpgB1 is important for secretion from bacteria and membrane targeting in eukaryotic cells. *PLoS One* **9**, e93461

11. Walhout, A. J., and Vidal, M. (2001) High-throughput yeast two-hybrid assays for large-scale protein interaction mapping. *Methods* **24**, 297-306
