# Supplementary material for: High-Throughput Screening of Type III Secretion Determinants Reveals a Major Chaperone-Independent Pathway
Source: mBio. 2018 Jun 19;9(3):e01050-18. doi: 10.1128/mBio.01050-18 (PMC6016238; doi:10.1128/mBio.01050-18)
Supplement: TABLE S5 [file mbo003183931st5.docx]

| **Table S5. Oligonucleotide summary.** | |
| --- | --- |
| **Oligo** | **Sequence, 5′–3′** |
| **5’ Oligos to generate deletion strains** | |
| IpgA_UP | CTCTCATTCTAATATATAGAAGGCCATAGAAATGTGTCGCGTGTAGGCTGGAGCTGCTTC |
| IpgE_5 Wanner | GGTGAAAGGGTATTCGTCATTTGTATAAGAGGAATATATGGTGTAGGCTGGAGCTGCTTC |
| Spa15_UP | TGTATTAAAGACTATTTAGTGAGGTTTAAATATGAGTAACGTGTAGGCTGGAGCTGCTTC |
| Spa47_5 Wanner | CTTATAATCAATGAGCTATACAAAATTGCTCACTCAATTAGTGTAGGCTGGAGCTGCTTC |
| **3’ Oligos to generate deletion strains** | |
| IpgA_DN | TGTTTAGAATTTGCATGATACCCCCTATATGTTAGTTCACCATATGAATATCCTCCTTAG |
| IpgE_3 Wanner | TAAATACGAAACGGGACATTAATACCCCTTCATTCTTCGCACATATGAATATCCTCCTTAG |
| Spa15_DN | ATTGAGTGAGCAATTTTGTATAGCTCATTGATTATAAGACCATATGAATATCCTCCTTAG |
| Spa47_3 Wanner | TTTTAAAACCTTATCTAATTGTTTCACCAATAAGCTCCATCATATGAATATCCTCCTTAG |
| **5’ Oligos to PCR *Shigella* ORFs into Gateway entry vectors** | |
| MxiL F GW | CGAAGGAGATAGAACCATGATTAATCAAATAAATGCAAGC |
| MxiA_952 F GW | CGAGGGGACAACTTTGTACAAAAAAGTTGGCATGGTCGTAGAAAAAGAAAAAAGTCTGTCTGAG |
| MxiC F GW | CGAAGGAGATAGAACCATGCTTGATGTTAAAAATAC |
| MxiE F GW | CGAAGGAGATAGAACC ATGAGTAAATATAAAGGTCTAAATAC |
| MxiG F GW | CGAGGGGACAACTTTGTACAAAAAAGTTGGCATGTCTGAGGCAAAGAACTCAAATCTTGCACC |
| MxiK F GW | CGAAGGAGATAGAACCATGATAAGAATGGATGGAATTTATA |
| MxiL F GW | CGAAGGAGATAGAACCATGATTAATCAAATAAATGCAAGC |
| MxiN F GW | CGAAGGAGATAGAACCATGAAGGTATGCAATATGCAAAAAG |
| OspI F GW | CGAAGGAGATAGAACCATGATTAATGGGGTGTCGTTACA |
| OspZ F GW | CGAAGGAGATAGAACCATGATTAGTCCCATCAAGAATATTA |
| Spa9 F GW | CGAGGGGACAACTTTGTACAAAAAAGTTGGCGTGTCTGATATAGTTTATATGGGTAATAAGGC |
| Spa13 F GW | CGAAGGAGATAGAACCATGGAGGCATTAGATAAAAGGATTA |
| Spa24 F GW | CGAGGGGACAACTTTGTACAAAAAAGTTGGCATGCTGAGTGACATGTCCCTCATCGC |
| Spa29 F GW | CGAGGGGACAACTTTGTACAAAAAAGTTGGCATGGACATTTCAAGCTGGTTCGAAAGTATTCATGTG |
| Spa32 F GW | CGAAGGAGATAGAACCATGGCATTAGATAATATAAA |
| Spa33 F GW | CGAAGGAGATAGAACCATGTGTGGGGATTGGGTAATTCGTA |
| Spa40 F GW | CGAAGGAGATAGAACCATGGCAAATAAAACAGAAAAGCCGA |
| Spa47 F GW | CGAAGGAGATAGAACCATGAGCTATACAAAATTGCTCACTC |
| Univ5 | GGGGACAACTTTGTACAAAAAAGTTGGCGAAGGAGATAGAACCATG |
| **3’ Oligos to PCR *Shigella* ORFs into Gateway entry vectors** | |
| MxiL R GW | GGGGACAACTTTGTACAAGAAAGTTGGTTACCATGTCGAATCATCTGCCTC |
| MxiA R GW | GGGGACAACTTTGTACAAGAAAGTTGGCTAAATAGTCTTTAATACATTAATGGTATATGC |
| MxiC R GW | GGGGACAACTTTGTACAAGAAAGTTGGTTATCTAGAAAGCTCTTTCT |
| MxiE R GW | GGGGACAACTTTGTACAAGAAAGTTGGTTAAATTTTTTCATTTATTTTTTTCACT |
| MxiG_378 R GW | GGGGACAACTTTGTACAAGAAAGTTGGCTACGAGTGGTTCTTATACATTCCGTTTAGTATATGGCC |
| MxiK R GW | GGGGACAACTTTGTACAAGAAAGTTGGTCATAGGCATGATGTCTGGATACCG |
| MxiL R GW | GGGGACAACTTTGTACAAGAAAGTTGGTTACCATGTCGAATCATCTGCCTC |
| MxiN R GW | GGGGACAACTTTGTACAAGAAAGTTGGTTAATCATTAACAGGATTCTCTTTT |
| OspI R GW | GGGGACAACTTTGTACAAGAAAGTTGGGCAAAGCCTCTTACTTTTCC |
| OspZ R GW | GGGGACAACTTTGTACAAGAAAGTTGGATAGACTTTAATCTCTGGCG |
| Spa9 R GW | GGGGACAACTTTGTACAAGAAAGTTGGTCAAACCCCACTCTTAATTAAAAACATTATTTCATGAC |
| Spa13 R GW | GGGGACAACTTTGTACAAGAAAGTTGGTTATCTAATGCCATACTTCATATCC |
| Spa24 R GW | GGGGACAACTTTGTACAAGAAAGTTGGCTAAGCAGGAATATTGATATATTGTTCAATCAAGGC |
| Spa29 R GW | GGGGACAACTTTGTACAAGAAAGTTGGTTATCTAACAAATAGATTTGTGAAAAATTTATGTTCACCG |
| Spa32 R GW | GGGGACAACTTTGTACAAGAAAGTTGGTTAGCATTCTTCTTCACTCT |
| Spa33 R GW | GGGGACAACTTTGTACAAGAAAGTTGGTTACTCCTTTACCATCCAAGAACTA |
| Spa40 R GW | GGGGACAACTTTGTACAAGAAAGTTGGTTAATGAGTGTTTTCAACCTGCTCA |
| Spa47 R GW | GGGGACAACTTTGTACAAGAAAGTTGGTCTAATTGTTTCACCAATAAGC |
| **Oligos used to generate MyoD fusion protein Gateway compatible destination vectors** | |
| OspB_50 R | GGGGACAACTTTGTACAAGAAAGTTGGTTTCTCTCCTAAAAAAACCAATGCT |
| OspB_100 R | GGGGACAACTTTGTACAAGAAAGTTGGACTCTGTGGTAGCCATGATTCAGC |
| OspB_200 R | GGGGACAACTTTGTACAAGAAAGTTGGAGCAGGGGCATTGTTAAAATTTTT |
| OspD1_50 R | GGGGACAACTTTGTACAAGAAAGTTGGTTCTTCATTGATGGCGTGGGATATA |
| OspD1_100 R | GGGGACAACTTTGTACAAGAAAGTTGGGTATTTACGCGCTGCATCGGCATT |
| OspD1_200 R | GGGGACAACTTTGTACAAGAAAGTTGGTGTAAGTTTTATCCCACTATGATG |
| OspF_50 R | GGGGACAACTTTGTACAAGAAAGTTGGATAGTACGCTGGGTATTGCCG |
| OspF_100 R | GGGGACAACTTTGTACAAGAAAGTTGGCCCAACAAAATCCCCCTTACTCTGA |
| OspF_200 R | GGGGACAACTTTGTACAAGAAAGTTGGAACATCTGACGCCGGATACTCCCCG |
| VirA_50 R | GGGGACAACTTTGTACAAGAAAGTTGGTTCGTGTGGAGAATATATGCCG |
| VirA_100 R | GGGGACAACTTTGTACAAGAAAGTTGGAAAAACCGAACATATGCCTTTGTT |
| VirA_200 R | GGGGACAACTTTGTACAAGAAAGTTGGTATTTTTGTACTATTGCTGTCAGA |
| DSW206 F | GACATCATAACGGTTCTGGC |
| RrnB R | GAAGAGCTCGTTTGTAGAAACGCAAAAAGGCC |
| **Oligos used to generate scanning deletion constructs** | |
| OspB_51_100_5 | TGGTTTTTTTAGGAGAGAAAGAACCAATAGTAATAAATAA |
| OspB_51_100_3 | TTATTTATTACTATTGGTTCTTTCTCTCCTAAAAAAACCA |
| OspB_101_150_5 | AATCATGGCTACCACAGAGTCAATTAGGCCTTGGTTCGGA |
| OspB_101_150_3 | TCCGAACCAAGGCCTAATTGACTCTGTGGTAGCCATGATT |
| OspB_151_200_5 | ACGGTAGTCCTGGTTCTCATGAAAGTCTTTCTTGTATCCT |
| OspB_151_200_3 | AGGATACAAGAAAGACTTTCATGAGAACCAGGACTACCGT |
| OspB_201_250_5 | ATTTTAACAATGCCCCTGCTCAAGAGCTTTTTCCCTACTC |
| OspB_201_250_3 | GAGTAGGGAAAAAGCTCTTGAGCAGGGGCATTGTTAAAAT |
| OspD1_51_100_5 | CCCACGCCATCAATGAAGAAAGTGAATCGCTGTTGGCAGC |
| OspD1_51_100_3 | GCTGCCAACAGCGATTCACTTTCTTCATTGATGGCGTGGG |
| OspD1_101_150_5 | CCGATGCAGCGCGTAAATACTTTGATTTATCACCAAAAGA |
| OspD1_101_150_3 | TCTTTTGGTGATAAATCAAAGTATTTACGCGCTGCATCGG |
| OspD1_151_200_5 | ATGGAGATTTTATTAAAACTGAAATAGCAGACAGACTTAA |
| OspD1_151_200_3 | TTAAGTCTGTCTGCTATTTCAGTTTTAATAAAATCTCCAT |
| OspF_50_150_5 | GGCAATACCCAGCGTACTATGACAAGTTTCATATTAGTAT |
| OspF_50_150_3 | ATACTAATATGAAACTTGTCATAGTACGCTGGGTATTGCC |
| OspF_100_150_5 | GTAAGGGGGATTTTGTTGGGGCTCAGTTTACGCTATATGT |
| OspF100_150_3 | ACATATAGCGTAAACTGAGCCCCAACAAAATCCCCCTTAC |
| OspF150_200_5 | AATCTCGTGTGGGGATAGGAAGACCTGAAGACTGGAAATA |
| OspF150_200_3 | TATTTCCAGTCTTCAGGTCTTCCTATCCCCACACGAGATT |
| VirA_50_150_5 | GCATATATTCTCCACACGAAGCACCAGAAGTTATTGAAAC |
| VirA_50_150_3 | GTTTCAATAACTTCTGGTGCTTCGTGTGGAGAATATATGC |
| VirA_150_250_5 | ATTTTAAAACTCACAAGCCTGACCTATGTTTTCAAACAGA |
| VirA_150_250_3 | TCTGTTTGAAAACATAGGTCAGGCTTGTGAGTTTTAAAAT |
| VirA_250_350_5 | ATTCAAACTTCCCCCAACAATCGCACATAACATGTAATGG |
| VirA_250_350_3 | CCATTACATGTTATGTGCGATTGTTGGGGGAAGTTTGAAT |
| **Oligos used to generate pmT3SA** | |
| minT3 1F | CGAGTACGGCCCCAAGGTCCAAACGGTGATTAATTTTTTAGTTGTTCATTTAATATCTG |
| minT3 2F | CCCTATATTACCCTGTTATCCCTAGCGTAACTATCGATCTCGAGTACGGCCCCAAGGTCC |
| minT3 3F | AATAGGCCGAAATCGGCAAGGATCCCTATATTACCCTGTTATCC |
| minT3 GBS R | TATACTTTAGATTTTAATTAAACGCGTTTAATTATCCTCAGTCATAAATGG |
