# Supplementary material for: High-Throughput Screening of Type III Secretion Determinants Reveals a Major Chaperone-Independent Pathway
Source: mBio. 2018 Jun 19;9(3):e01050-18. doi: 10.1128/mBio.01050-18 (PMC6016238; doi:10.1128/mBio.01050-18)
Supplement: Supplementary file 6 [file mbo003183931st3.docx]

Table S3. Summary of Y2H studies.

GAL4 binding domain (BD) fusions	GAL4 activation domain (AD) fusions																
	IpaA	IpaH1.4	IpaH4.5	IpaH7.8	IpaH9.8	IpgB1	OspB	OspC1	OspC2	OspC3	OspD1	OspD2	OspD3	OspE	OspF(K134A)	OspG	OspZ
IpgF	No	No	No	No	No	No	No	No	No	No	No	No	No	No	No	No	No
MxiA(318-686)	No	No	No	No	No	No	No	No	No	No	No	No	No	No	No	No	No
MxiC	No	No	No	No	No	No	No	No	No	No	No	No	No	No	No	No	No
MxiE	No	No	No	No	No	No	No	No	No	No	No	No	No	No	No	No	No
MxiG(1-126)	No	No	No	No	No	No	No	No	No	No	No	No	No	No	No	No	No
MxiK	No	No	No	No	No	No	No	No	No	No	No	No	No	No	No	No	No
MxiL	No	No	No	No	No	No	No	No	No	No	No	No	No	No	No	No	No
MxiN	No	No	No	No	No	No	No	No	No	No	No	No	No	No	No	No	No
Spa9	No	No	No	No	No	No	No	No	No	No	No	No	No	No	No	No	No
Spa13	No	No	No	No	No	No	No	No	No	No	No	No	No	No	No	No	No
Spa24	No	No	No	No	No	No	No	No	No	No	No	No	No	No	No	No	No
Spa29	No	No	No	No	No	No	No	No	No	No	No	No	No	No	No	No	No
Spa32	No	No	No	No	No	No	No	No	No	No	No	No	No	No	No	No	No
Spa33	No	No	No	No	No	No	No	No	No	No	No	No	No	No	No	No	No
Spa40	No	No	No	No	No	No	No	No	No	No	No	No	No	No	No	No	No
Spa47	No	No	No	No	No	No	No	No	No	No	No	No	No	No	No	No	No
Spa15	Yes	Yes	No	No	No	Yes	Yes	Yes	Yes	Yes	Yes	Yes	No	No	No	No	No
